# Early Perioperative Changes in Circulating Hypoxia-Inducible Factor-1 Alpha (HIF-1A) and Vascular Endothelial Growth Factor (VEGF) Following Lower Limb Revascularisation for Peripheral Arterial Disease

**DOI:** 10.3390/jcm15114288

**Published:** 2026-06-01

**Authors:** Benedek Kasza, Gábor Kasza, Tibor Nagy, Gábor Jancsó, Ibitamuno Caleb

**Affiliations:** 1 Department of Translational Medicine, Medical School, University of Pécs, Szigeti Street 12, H-7624 Pécs, Hungary; kasza.benedek@pte.hu; 2 Department of Vascular Surgery, Clinical Centre and Medical School, University of Pécs, Ifjúság Street 13, H-7624 Pécs, Hungary

**Keywords:** peripheral arterial disease (PAD), chronic limb ischaemia, intermittent claudication (IC), ischaemia–reperfusion injury (IRI), hypoxia, lower limb bypass surgery, femoropopliteal bypass

## Abstract

**Background**: Peripheral arterial disease (PAD) is characterised by chronic limb ischaemia and frequently coexists with metabolic comorbidities like diabetes mellitus. Surgical revascularisation performed to relieve symptoms of ischaemia induces an abrupt transformation from hypoxia toward normoxia. However, the early systemic behaviour of hypoxia-responsive angiogenic pathways during this period remains incompletely characterised. **Methods**: In this prospective, single-centre observational study, 26 patients undergoing elective above-knee femoropopliteal bypass for PAD were enrolled. Venous samples were collected preoperatively (T0), immediately postoperatively (T1), and 24 h after surgery (T2). Circulating hypoxia-inducible factor-1 alpha (HIF-1A) and vascular endothelial growth factor (VEGF) were quantified, and their temporal changes were analysed. Exploratory subgroup analyses were also performed according to diabetes status. **Results**: Both circulating HIF-1A and VEGF declined significantly following revascularisation, with reductions detectable immediately postoperatively and persisting at 24 h. Baseline HIF-1A and VEGF concentrations were strongly correlated. While baseline biomarker levels did not differ by diabetes status, patients with diabetes exhibited an attenuated postoperative decline in circulating HIF-1A at 24 h, with a similar but non-significant trend observed for VEGF. **Conclusions**: Lower limb revascularisation for PAD is associated with a rapid and sustained decline in circulating hypoxia-responsive angiogenic markers, consistent with early resolution of hypoxia-driven signalling. Diabetes status was associated with altered postoperative biomarker trajectories, supporting further investigation of early perioperative angiogenic dynamics in PAD.

## 1. Introduction

Peripheral arterial disease (PAD) of the lower limb is characterised by progressive atherosclerotic narrowing and occlusion of arterial vessels, ultimately impairing blood flow and resulting in chronic limb ischaemia [1,2]. It remains a major cause of morbidity worldwide and commonly develops in the context of systemic metabolic and vascular dysfunction, including diabetes mellitus, hypertension, smoking-related endothelial injury, and chronic low-grade inflammation [1,2]. Advanced disease can markedly impair quality of life, with clinical manifestations ranging from exercise-limiting intermittent claudication to rest pain, tissue loss, and gangrene [2,3].

PAD is associated not only with large-vessel obstruction but also with structural and functional disturbances at the microvascular and cellular level [4]. Prolonged arterial narrowing results in a state of chronic tissue hypoxia, which activates adaptive signalling pathways involved in angiogenesis, cellular metabolism, and survival. Central to this response is hypoxia-inducible factor-1 alpha (HIF-1A), a transcription factor that coordinates cellular adaptation to reduced oxygen availability, including induction of vascular endothelial growth factor (VEGF) [5,6]. Experimental and clinical studies have demonstrated upregulation of the HIF-1A–VEGF axis in ischaemic tissues, supporting its role in angiogenic and reparative processes [5,7].

In advanced cases of PAD, surgical or endovascular revascularisation is required to restore arterial inflow, alleviate ischaemic symptoms, promote wound healing, and prevent major amputation [1,2]. While revascularisation improves macroscopic blood flow, its immediate systemic biological consequences remain an active area of investigation [8,9,10]. The abrupt transition from prolonged hypoperfusion to restored oxygen delivery initiates a complex cascade known as ischaemia–reperfusion injury (IRI), characterised by oxidative stress, inflammatory signalling, endothelial activation, and changes in circulating mediators, all of which may influence hypoxia-inducible pathways in the early postoperative period [8,11]. Previous studies have explored selected circulating biomarkers as indicators of perioperative physiological responses to IRI [7,8]. However, measurable changes in key components of hypoxia-responsive angiogenic pathways during the early stages of reperfusion of PAD patients has received limited attention. Most available PAD studies have focused either on tissue-level molecular changes or on later postoperative timepoints [6,10], whereas the immediate systemic behaviour of circulating HIF-1A and VEGF after restoration of blood flow remains insufficiently characterised.

Furthermore, in PAD patients, metabolic comorbidities, particularly diabetes mellitus (DM), are common, and have been associated with impaired endothelial function, reduced angiogenic capacity, and altered cellular responses to hypoxia, potentially contributing to delayed recovery following revascularisation [12,13,14]. Previous studies suggest that DM may modulate hypoxia-responsive signalling, in part through effects on HIF-1A stability and VEGF responsiveness [13,14,15]. However, the impact of diabetes on the early perioperative behaviour of hypoxia-related biomarkers in PAD has not been well characterised. Particularly, it remains unclear whether diabetes status is associated with any immediate difference in the measurable systemic response of the HIF-1A–VEGF axis following surgical reperfusion, compared with patients without diabetes.

Accordingly, this study aimed to characterise early perioperative changes in circulating HIF-1A and VEGF in patients undergoing open lower limb revascularisation for PAD, with particular focus on the immediate postoperative period. In addition, we explored whether diabetes status was associated with differences in these biomarker trajectories.

## 2. Methods

### 2.1. Study Design and Population

This prospective, single-centre observational study enrolled 26 consecutive patients undergoing elective above-the-knee (AK) femoropopliteal bypass surgery for peripheral arterial disease at the Department of Vascular Surgery of the University of Pécs Clinical Centre, Pécs, Hungary, between February and June 2022. These patients presented with severe, life-limiting intermittent claudication (Fontaine class IIb). Digital Subtracting Angiography (DSA) was the imaging of choice to identify the location of arterial stenosis.

Given that PAD patients are a very heterogenous group, we specifically selected patients in whom the severe stenosis was found in the superficial femoral artery (SFA) and for whom an AK femoropopliteal bypass was indicated. Although some patients had additional lesions beyond the SFA these were not considered clinically significant to warrant further planned intervention and hence were not characterised. Surgical lower limb revascularisation was used as a controlled human model of ischaemia–reperfusion occurring on a background of chronic metabolic and vascular dysfunction. Restricting the cohort to patients undergoing AK femoropopliteal bypass for similar anatomical disease patterns allowed us to reduce procedural heterogeneity while examining systemic hypoxia–angiogenic responses in a population enriched for metabolic comorbidities. The decision to operate was made independently by the treating physician, without any influence from the study investigators.

All procedures were performed under general anaesthesia by experienced vascular surgeons using standard techniques for above-the-knee (AK) femoropopliteal bypass. Briefly, the common femoral artery (CFA) and above-knee popliteal artery (PA) were exposed. Thereafter, proximal, and distal control was obtained, and the time of clamping was noted. Revascularisation was performed using either autologous great saphenous vein or a prosthetic Dacron graft. The choice of the graft used was purely a clinical decision made by the treating physician. Generally, great saphenous vein (GSV) grafts were preferentially used when suitable. Prosthetic grafts (Dacron) were employed in case of inadequate or absent GSV. After successful anastomosis (end-to-side technique), the vessels were unclamped to restore perfusion. Finally, adequate bleeding control was performed, before surgical drain insertion and closure. Perioperative anaesthesia, anticoagulation, and fluid management followed institutional standard protocols and were not modified for study purposes.

Inclusion criteria were age ≥ 18 years, life-limiting intermittent claudication (Fontaine class IIb), severe stenosis found in the superficial femoral artery (SFA), elective open surgical revascularisation using autologous vein or prosthetic (Dacron) graft, and ability to provide written informed consent. Exclusion criteria included acute limb ischaemia, active infection, non-healing ulcers or gangrene and severe comorbidities precluding surgery. The study was approved by the Regional Committee for Research Ethics of the University of Pécs, Hungary (No. 8914–PTE 2021).

### 2.2. Clinical Data Collection

Baseline demographics, comorbidities (diabetes mellitus, smoking status, chronic kidney disease [eGFR < 60 mL/min/1.73 m^2^]), Fontaine category, and operative details were recorded. Surgery time, defined as the time from skin incision to skin closure, and clamp time which reflects the period under which the artery was under cross clamping were recorded for each patient. Diabetes was defined per clinical history or antidiabetic therapy. Current smoking was self-reported. Adverse limb events (ALE) within 3 months were recorded using predefined criteria: graft occlusion requiring reintervention, minor or major amputation.

### 2.3. Biomarker Sampling

Peripheral venous blood samples were collected at three time points:

T0: Preoperatively;

T1: Immediately postoperatively (within 30 min of completion of the surgery, i.e., post-skin closure);

T2: 24 h postoperatively.

Samples were centrifuged (10 min at 1500 rcf.), and plasma was immediately stored at −80 °C, thawed only once and analysed on the same day. Haemolysis-free samples were prioritised.

### 2.4. Biomarker Assays

Plasma HIF-1A was measured using a commercially available human HIF-1A ELISA kit (ABclonal, ABClonal Germany GmbH, Düsseldorf, Germany), validated for quantitative measurement in human serum, plasma, cell culture supernatant, and other biological fluids. The assay had a reported dynamic range of 78.13–5000 pg/mL and a minimum detectable dose of <35.1 pg/mL. According to the manufacturer, the intra- and inter-assay coefficients of variation were <10% and <15%, respectively; manufacturer validation data reported intra-assay CVs of 2.1–3.3% and inter-assay CVs of 4.3–6.8%. Plasma VEGF was measured using a human VEGF ELISA kit (Biomedica Immunoassays, Biomedica Hungaria Ltd., Budapest, Hungary), with a reported assay range of 0–2000 pg/mL, a limit of detection of 2.5 pg/mL, a lower limit of quantification of 15.6 pg/mL, a within-run coefficient of variation of ≤3%, and a between-run coefficient of variation of ≤6%. Samples were processed according to the manufacturer’s instructions.

Each sample was assayed in duplicate, and the mean value was used for analysis. Absorbance at 450 nm was recorded using a microplate reader that had been zeroed against the blank wells, ensuring that all reported optical density (OD) values represented background-corrected absorbance. These OD readings were converted to concentrations (pg/mL) using the standard curves generated from the assay calibrators. In addition, plasma albumin (Alb, g/L) was measured using a Cobas 8000 Modular Analyzer (Roche Diagnostics, GmbH, Mannheim, Germany) following the manufacturer’s instructions [16].

### 2.5. Statistical Analysis

Data visualisation and statistical analyses were performed using Microsoft Excel for Microsoft 365 MSO (version 2308) and GraphPad Prism (version 8.0). Both the raw plasma values of HIF-1A and VEGF, and their values normalised to albumin (HIF1A/Alb, VEGF/Alb respectively) were analysed. Albumin normalisation was performed to partially account for the haemodilution from perioperative fluid shifts and capillary leak, which commonly reduce plasma protein concentrations during surgery [17].

Due to limited preliminary data on this topic, no formal sample size calculation was performed. Accordingly, subgroup analyses were exploratory and intended for hypothesis generation.

Continuous variables are presented as median (interquartile range, IQR) and categorical data are *n* (%). Due to the small sample size of the study, non-parametric methods were applied. Changes in HIF-1A and VEGF concentrations across the three time points (baseline (T0), immediately after revascularisation (T1), and at 24 h (T2)) were evaluated using the Friedman test for repeated measures. When the Friedman test indicated a significant overall difference, pairwise time-point comparisons were performed using Dunn’s post hoc test. Correlation analysis was performed using Spearman’s correlation.

For calculating the magnitude of change (%Δ) in the markers for each patient the following formula was used: %Δ for immediate postoperative response (T1%Δ): [(T1 − T0)/T0] × 100. For the 24 h postoperative response (T2%Δ): [(T2 − T0)/T0] × 100. A negative (−) sign in front of the value signifies a downward change (reduction), while a positive (+) sign in front of the value signifies an upward change (increase).

Statistical significance was defined as *p* < 0.05. Data are reported as medians with interquartile ranges unless otherwise specified.

## 3. Results

### 3.1. Patient Characteristics

Twenty-six patients undergoing elective above-the-knee (AK) femoropopliteal bypass surgery for lower limb peripheral arterial disease (PAD) were included. The median age was 68 years; 69% of the patient were male. Diabetes mellitus was present in 42% and for the majority of the patients, the graft of choice was the greater saphenous vein (GSV) graft (Table 1).

### 3.2. Overall Biomarker Kinetics

**Figure 1:**
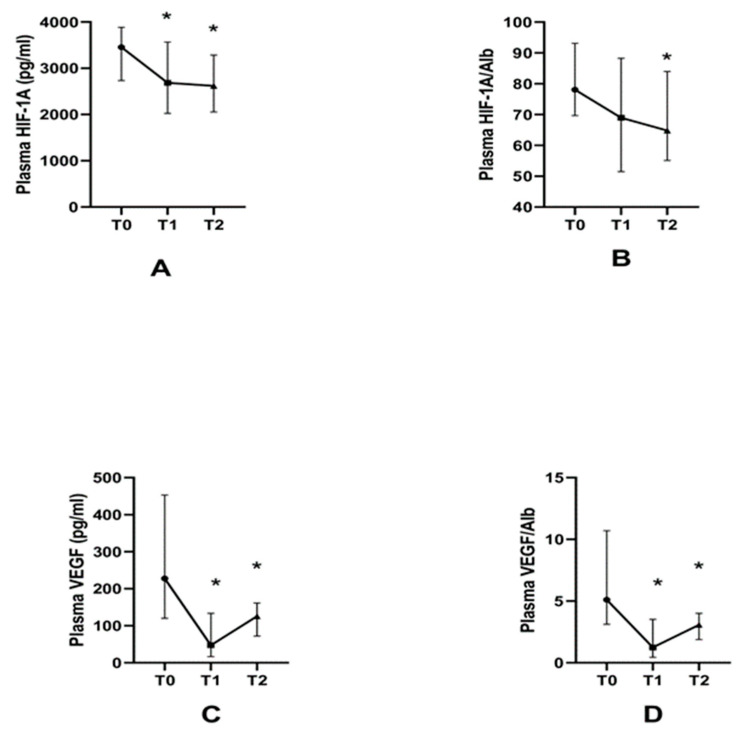
(**A**–**D**). Changes from baseline in plasma HIF-1A and VEGF levels. Panels (**A**,**B**) show changes in plasma HIF-1A concentrations with and without normalisation to albumin (Alb), respectively. Panels (**C**,**D**) show corresponding raw and albumin-normalised plasma VEGF concentrations. * Indicates a significant difference from the baseline values (T0).

Plasma HIF-1A and VEGF decreased significantly from baseline (Figure 1). HIF-1A fell immediately postoperatively (T1: 2686 (2024–3565) vs. T0: 3456 (2736–3886) pg/mL; *p* = 0.0368) and remained low at 24 h (T2: 2617 (2053–3284) pg/mL; *p* = 0.0022 vs. T0). VEGF showed steeper immediate reduction after revascularisation (T1: 47.33 (16.44–133.60) vs. T0: 227.60 (120.20–453.30) pg/mL; *p* < 0.0001) with partial recovery at T2 (125.80 (71.95–161.60) pg/mL vs. T0; *p* = 0.0011) (Figure 1).

This pattern was maintained even after adjustment with albumin values for HIF-1A (T1: 68.96 (51.50–88.28) vs. T0: 78.06 (69.68–93.12), *p* = 0.331; T2: 64.82 (55.15–83.96), *p* = 0.011) and for VEGF (T1: 1.25 (0.45–3.52) vs. T0: 5.11 (3.11–10.71), *p* < 0.0001; T2: 3.10 (1.88–4.00), *p* = 0.002) respectively (Figure 1).

These results demonstrate an immediate decline in hypoxia–angiogenic markers after revascularisation surgery.

**Figure 2:**
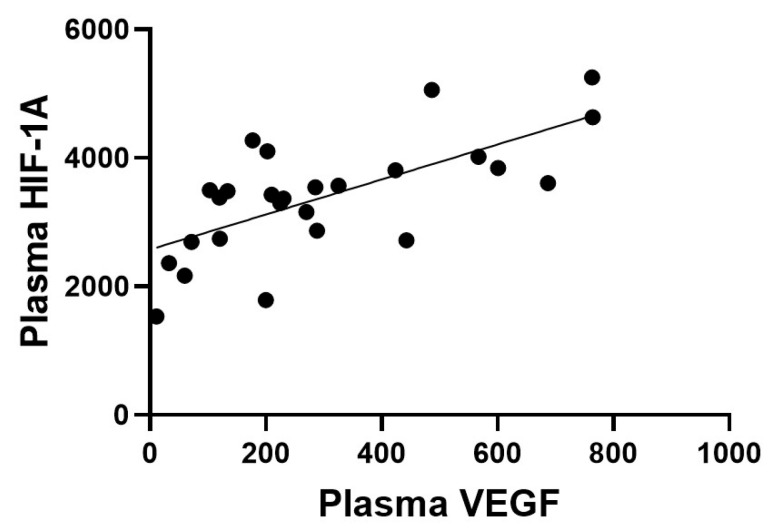
Scatter plot showing a positive linear correlation between plasma HIF-1A and plasma VEGF.

Furthermore, baseline HIF-1A and VEGF showed strong correlation (r = 0.67, *p* = 0.0002) (Figure 2). Immediate %ΔT1 values also showed moderate coupling (r = 0.54, *p* = 0.004), and at 24 h (r = 0.49, *p* = 0.011) (Figure 2). These results support the association between these two hypoxia-sensitive markers.

### 3.3. Diabetes Subgroup Analysis

Eleven patients in this study presented with diabetes mellitus (DM). There was no difference in median surgery time (181.9 (168.0–191.0) min vs. 170.2 (159.0–185.0) min, *p* = 0.18) or clamp time for patients (32.0 (29.0–35.0) min vs. 30.0 (28.0 vs. 34.0) mins, *p* = 0.36) with DM or without DM respectively. The choice of graft was also numerically comparable between the groups (DM: 8/11 for GSV graft, 3/11 for Dacron graft, NoDM: 8/15 for GSV graft, 7/15 for Dacron graft).

**Figure 3:**
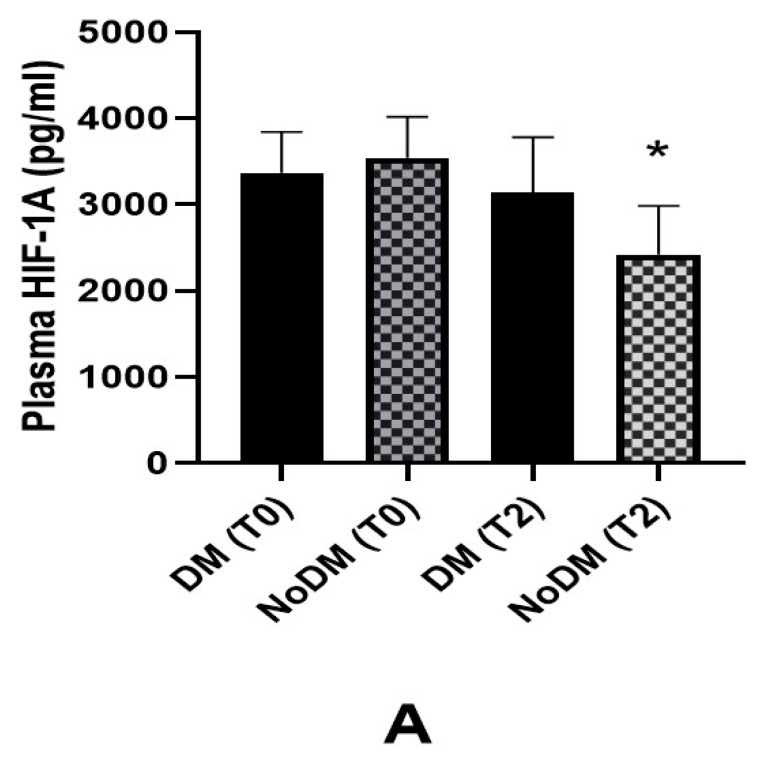

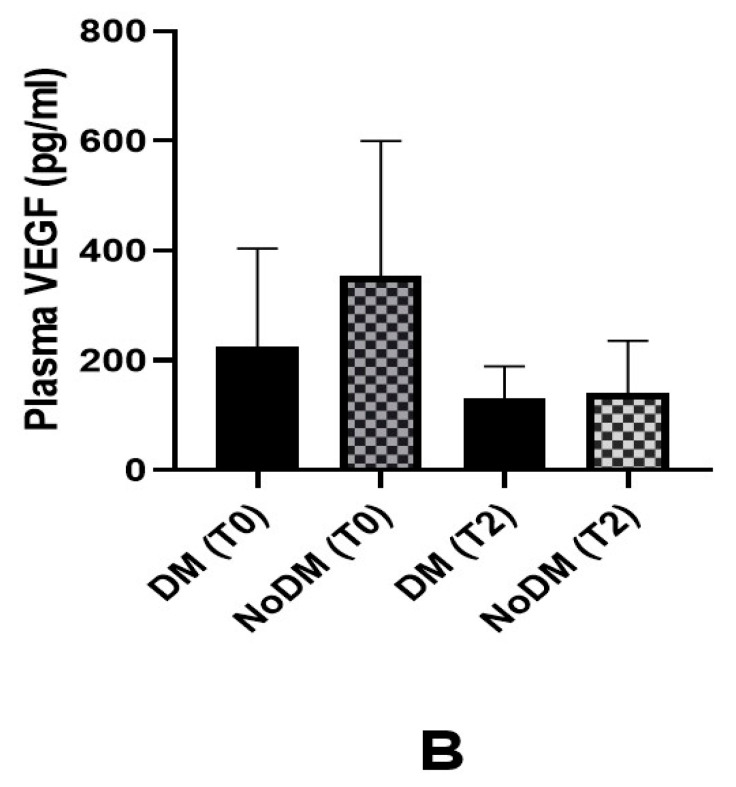
(**A**,**B**) Comparison of changes in median plasma HIF-1A (**A**) and VEGF (**B**) concentrations between patients with diabetes (DM) and those without diabetes (NoDM) at two time points: pre- (T0) and post- (T2) revascularisation. * Indicates a significant difference between DM and NoDM groups at the corresponding time point.

At baseline (T0), there was no significant difference between plasma HIF-1A in patients with DM vs. patients without DM (DM-HIF1A: 3369 (2363–3842) vs. NoDM-HIF1A: 3544 (2741–4017), *p* = 0.384). However, after revascularisation at T2, the median values of HIF1A were significantly higher in patients with DM (3142 (2635–3783)) vs. those without DM (2419 (1898–2985)), *p* = 0.003 (Figure 3A).

VEGF also exhibited a similar pattern of no difference at the baseline level (DM-VEGF: 199.50 (119.70–269.60) vs. NoDM-VEGF: 228.10 (120.40–566.80), *p* = 0.198). Here, as well as a day after revascularisation (T2), DM patients had higher plasma VEGF values than non-DM patients; however, it was not significant in this case (109.00 (76.62–156.60) vs. 128.10 (62.47–169.80), respectively, *p* = 0.8785) (Figure 3B).

The magnitude of change was also compared between these two groups. Here patients with DM exhibited significantly blunted suppression of HIF-1A vs. non-diabetic patients (−2.86% (−11.45–+32.98) vs. −27.96% (−37.23–−24.33); respectively. *p* < 0.001). VEGF trends were directionally similar but non-significant (−35.97% (−52.18–−18.30) vs. –44.26% (−71.05–−35.86), respectively, *p* = 0.11).

Taken together these results show that in DM there is a blunted reduction from baseline values of HIF-1A and VEGF in circulation after revascularisation compared to those without DM.

### 3.4. Adverse Limb Events (Exploratory Analysis)

There were no recorded adverse limb events (ALEs) during the period of hospital admission for any of the patients. ALEs occurred in five patients (19%) within 3 months. In four out of five of these patients graft occlusion needing mechanical thrombectomy using a Fogarty catheter was recorded, while one patient received a below-the-knee amputation due to a fatal ischaemic event within the follow-up period.

As an exploratory measure, we retrospectively compared the average of baseline HIF-1A and VEGF markers and the magnitude of change between these patients with ALEs vs. those without. At baseline (T0), patients who developed ALEs had slightly lower values of HIF-1A compared to those who did not develop ALEs (2923.3 (2216.4–4344.3) vs. 3461.5 (3085.3–3814.8)). The VEGF values in ALE patients also showed lower values compared to non-ALE (96.5 (39.1–398.0) vs. 250.0 (193.8–428.3)).

The magnitude of reduction from baseline at T2 was also compared between ALE and non-ALE patients. In ALE patients, the HIF-1A values tended to show a blunted suppression of this marker in the plasma compared to non-ALE (+10.7% (−22.3–+37.0) vs. −22.9% (−30.4–−10.4)). This pattern was also observed for the VEGF values with ALE patients also showing similar comparatively high values (−27.1% (−44.50–+112.1) vs. −43.5 (−60.9–−35.9)).

Taken together these results illustrate possible differences in perioperative kinetics of HIF-1A and VEGF in patients who developed ALEs compared to those who did not.

## 4. Discussion

In this present study, we examined early perioperative changes in circulating hypoxia-responsive angiogenic markers in patients undergoing lower limb revascularisation for peripheral arterial disease (PAD). PAD represents a state of chronic arterial insufficiency that frequently occurs in the presence of metabolic comorbidity [2]. Our results show that restoration of arterial inflow was associated with a rapid decline in circulating HIF-1A and VEGF concentrations, evident within minutes after surgery and sustained at 24 h. This temporal pattern remained broadly consistent after normalisation to albumin, suggesting that the observed changes were less likely to be explained by perioperative fluid shifts alone.

From a biological perspective, the early reduction in circulating HIF-1A is consistent with known oxygen-dependent regulation of this hypoxia-sensitive protein. Following reperfusion, improved tissue oxygen tension promotes prolyl-hydroxylase domain (PHD) enzymes-mediated ubiquitination and proteasomal degradation of HIF-1A. This process can occur rapidly, within minutes of reoxygenation [18,19]. Although relief of tissue hypoxia following revascularisation provides a consistent and plausible explanation for the observed decline in circulating HIF-1A at T1, measured levels likely represent the net effect of competing physiological processes. These include oxygen-driven protein degradation alongside persistent contributions from vascular stress, endothelial injury, immune activation, and early cellular turnover associated with the ischaemia–reperfusion injury (IRI) cascade [20,21].

Furthermore, the concurrent reduction in circulating VEGF may reflect the downstream attenuation of hypoxia-driven angiogenic signalling. However, like HIF-1A, systemic VEGF levels may additionally be influenced by endothelial activation, platelet dynamics, and inflammatory stimuli associated with surgical intervention and IRI [22,23].

From the design of this present study, it is not possible to determine to what extent the early decline in circulating HIF-1A and VEGF reflects hypoxia-driven suppression or the influence of oxidative and inflammatory processes within the IRI cascade. Nevertheless, the observed pattern is consistent with previous intramuscular and circulating studies reporting reductions in these markers following revascularisation [6,10,24]. Our findings extend this literature by demonstrating that this decline is already detectable within a very early postoperative timeframe. Furthermore, the persistence of reduced plasma levels of these marker proteins at 24 h after revascularisation suggests that the observed changes may largely reflect the haemodynamic effects of revascularisation, including restoration of oxygenated blood flow to the previously ischaemic limb [6,10].

Emerging evidence from experimental and clinical studies indicates that metabolic diseases like diabetes alter vascular responses to ischaemia–reperfusion (IR) beyond changes in large-vessel blood flow [25]. In diabetes, microvascular damage and endothelial dysfunction contribute to impaired tissue recovery post-revascularisation [25]. Differences in hypoxia-related protein trajectories post-reperfusion have been observed in other organs [26,27]. Given that diabetes is a frequent comorbidity in PAD patients, which has been linked to worsening outcomes in revascularised patients [28], we investigated potential differences in the trajectory of hypoxia-associated markers after revascularisation.

From our results, while baseline circulating HIF-1A and VEGF concentrations were comparable between patients with and without diabetes, individuals with diabetes exhibited a less pronounced decline in circulating HIF-1A at 24 h following revascularisation. A similar but non-significant trend was also observed for VEGF. These findings suggest that diabetes may modify the normalisation of hypoxia-responsive signalling after reperfusion, despite restoration of macroscopic arterial blood flow.

Several non-mutually exclusive mechanisms may underlie this observation. Diabetes is associated with endothelial dysfunction, oxidative stress, and microvascular impairment, factors that may influence oxygen delivery and cellular oxygen sensing after revascularisation [27,29]. Furthermore, studies show that chronic hyperglycaemia can also alter PHD activity, which in turn can dysregulate HIF-1A response despite restoration of oxygen delivery [29,30]. Within this context, attenuated suppression of circulating HIF-1A may reflect persistent microvascular dysfunction, delayed normalisation of tissue stress responses, or altered systemic handling of hypoxia-responsive mediators.

Alternatively, the comparatively reduced decline in these hypoxia-sensitive proteins in patients with DM compared to those without may represent an adaptive or compensatory response in tissues with an impaired vascular reserve. Several studies have ascribed therapeutic benefit to augmented HIF-1A activity in promoting angiogenesis and enhancing wound healing in diabetic settings [31,32]. Within this framework, incomplete downregulation of HIF-1A following revascularisation may reflect an adaptive mechanism aimed at sustaining reparative signalling. However, considering the observational design and limited sample size of the present study, these interpretations should be appraised cautiously.

A further possible explanation for the different hypoxia-related biomarker trajectories observed between the DM and non-DM patients is that DM is widely associated with a more distal, below-the-knee (infrapopliteal) pattern of atherosclerotic stenosis [28]. In this cohort, although the main lesion was in the superficial femoral artery (SFA), we cannot exclude a contribution from concomitant below-the-knee disease to the reperfusion response. However, because a quantitative assessment of infrapopliteal disease burden and between-group comparison of findings were beyond the scope of this study, this issue could not be further explored. These considerations may form the basis of future studies.

Exploratory analysis of adverse limb events (ALEs) suggested that patients who later developed limb-related complications tended to show attenuated early postoperative reductions in circulating HIF-1A and VEGF. However, the small number of events precludes any meaningful statistical inference; therefore, these observations should be regarded as hypothesis-generating only. Nevertheless, this pattern is consistent with previous reports indicating that perioperative angiogenic profiles may differ between patients with favourable and unfavourable vascular outcomes following revascularisation [6,24,33]. For example, Schönborn et al. reported that patients who remained free of adverse events after lower limb revascularisation exhibited a distinct postoperative angiogenic profile during follow-up compared with those who later developed complications [24]. Given the clinical need for easily accessible biomarkers that may help predict limb recovery or adverse outcomes in PAD, early postoperative profiling of angiogenic proteins may represent a potential avenue for further investigation. Whether early hypoxia-responsive biomarker dynamics have true prognostic value in PAD, however, will require confirmation in larger, adequately powered studies.

Beyond their descriptive value, the present findings have potential implications for therapeutic strategies. Numerous clinical trials of VEGF-based or HIF-targeted gene therapies have failed to produce significant benefit in PAD [5,34]. One possible explanation is that the optimal biological window for augmenting angiogenesis is still poorly defined [35]. The early, rapid decline in circulating HIF-1A and VEGF observed in our study suggests that interventions targeting these pathways may need to be precisely timed relative to the revascularisation process. Failure to consider this postoperative biology may partially explain prior negative trials, and future strategies might benefit from aligning therapeutic delivery with periods where they may have optimal biological impact.

This study has several limitations. The sample size was modest, particularly for subgroup analyses, which limits the power to detect smaller between-group differences. Therefore, these should be considered as hypothesis-generating.

Furthermore, although circulating angiogenic biomarkers are clinically practical and widely used, they do not fully capture tissue level gradients of hypoxia or microvascular perfusion and therefore provide only an indirect view of local ischaemic physiology. This is particularly relevant for HIF-1A, whose detection in circulation, despite being an intracellular protein, remains a useful biomarker of hypoxia-related biology [6,20,21]. Nevertheless, its plasma concentration may be influenced by cellular turnover or injury, extracellular vesicle-associated release, and systemic IRI-related processes, and should therefore be interpreted in that context [20,21]. Hence, incorporating direct assessments, such as muscle biopsies, perfusion imaging, or microvascular functional testing, would offer a more comprehensive understanding of these processes.

Nevertheless, restricting the cohort to PAD patients with similar patterns of arterial occlusion who underwent the same revascularisation procedure reduces clinical heterogeneity and strengthens the internal validity of the observations [10]. Moreover, despite the inherent constraints, the observed temporal patterns were consistent with known physiological responses, which aids in interpreting the results.

## 5. Conclusions

In conclusion, limb revascularisation by means of femoropopliteal bypass in PAD patients is associated with a rapid and sustained fall in circulating HIF-1A and VEGF. Furthermore, DM, as a common metabolic comorbidity in PAD, was associated with altered postoperative biomarker trajectories, suggesting that systemic metabolic disease may influence molecular responses to reperfusion. These observations contribute to a more nuanced understanding of perioperative angiogenic vascular biology in PAD and support further investigation aimed at their relevance for therapeutic approaches and risk stratification strategies.

## Figures and Tables

**Table 1:** Baseline characteristics of patients presenting for above-the-knee (AK) femoropopliteal bypass surgery.

Patient Variable	N = 26
Sex (male) *n* (%)	18 (69)
Age (years)	68 (62–74)
Diabetes Mellitus *n* (%)	11 (42)
Hypertension *n* (%)	21 (81)
Ischaemic Heart Disease *n* (%)	9 (35)
Smoker *n* (%)	17 (65)
Dyslipidaemia *n* (%)	10 (38)
Chronic Kidney Disease *n* (%)	7 (26)
Greater Saphenous Vein Graft (GSV) *n* (%)	16 (62)
Dacron Graft *n* (%)	10 (38)
Surgery Time (minutes)	174.0 (161.5–187.3)
Clamp Time (minutes)	31.0 (28.0–38.0)

Percentages (%) which represent the proportion to the whole are rounded up to the nearest whole number. Continuous variables are presented as median (interquartile range).

## Data Availability

Data are available on reasonable request to the corresponding author.

## Abbreviations

The following abbreviations are used in this manuscript:

PAD	Peripheral Arterial Disease
HIF-1A	Hypoxia-Inducible factor-1 Alpha
VEGF	Vascular Endothelial Growth Factor
IRI	Ischaemia–Reperfusion Injury
